# Rapid Pacing Decreases L-type Ca^2+^ Current and Alters *Cacna1c* Isogene Expression in Primary Cultured Rat Left Ventricular Myocytes

**DOI:** 10.1007/s00232-023-00284-y

**Published:** 2023-03-30

**Authors:** Anne Ritzer, Tobias Roeschl, Sandra Nay, Elena Rudakova, Tilmann Volk

**Affiliations:** 1 Institut für Zelluläre und Molekulare Physiologie, Friedrich-Alexander-Universität Erlangen-Nürnberg, Waldstraße 6, 91054 Erlangen, Germany; 2 Muscle Research Center Erlangen (MURCE), Friedrich-Alexander-Universität Erlangen-Nürnberg, 91054 Erlangen, Germany

**Keywords:** *Cacna1c*, Cardiac memory, Regional differences, Remodeling

## Abstract

The L-type calcium current (I_CaL_) is the first step in cardiac excitation–contraction-coupling and plays an important role in regulating contractility, but also in electrical and mechanical remodeling. Primary culture of cardiomyocytes, a widely used tool in cardiac ion channel research, is associated with substantial morphological, functional and electrical changes some of which may be prevented by electrical pacing. We therefore investigated I_CaL_ directly after cell isolation and after 24 h of primary culture with and without regular pacing at 1 and 3 Hz in rat left ventricular myocytes. Moreover, we analyzed total mRNA expression of the pore forming subunit of the L-type Ca^2+^ channel (*cacna1c*) as well as the expression of splice variants of its exon 1 that contribute to specificity of I_CaL_ in different tissue such as cardiac myocytes or smooth muscle. 24 h incubation without pacing decreased I_CaL_ density by ~ 10% only. Consistent with this decrease we observed a decrease in the expression of total *cacna1c* and of exon 1a, the dominant variant of cardiomyocytes, while expression of exon 1b and 1c increased. Pacing for 24 h at 1 and 3 Hz led to a substantial decrease in I_CaL_ density by 30%, mildly slowed I_CaL_ inactivation and shifted steady-state inactivation to more negative potentials. Total *cacna1c* mRNA expression was substantially decreased by pacing, as was the expression of exon 1b and 1c. Taken together, electrical silence introduces fewer alterations in I_CaL_ density and *cacna1c* mRNA expression than pacing for 24 h and should therefore be the preferred approach for primary culture of cardiomyocytes.

**Graphical Abstract:**

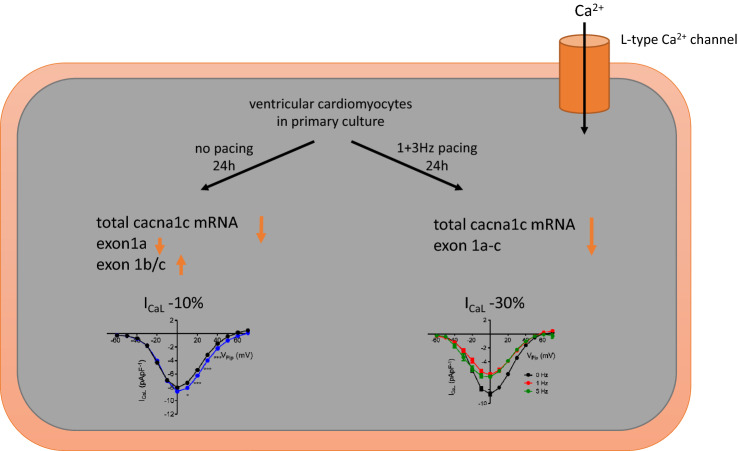

## Introduction

### Function of L-type Calcium Channel in the Heart and Alternative Splicing

The L-type Ca^2+^ current (I_CaL_) contributes to shape and duration of the ventricular action potential (AP) in the heart of many species including rodents, canine and man (Coraboeuf and Nargeot 1993; Linz and Meyer 2000). Moreover, Ca^2+^ influx via the L-type Ca^2+^ channel is the first step in the excitation–contraction-coupling cascade, its magnitude controls the amount of Ca^2+^ released from the sarcoplasmic reticulum (SR) and contributes to the filling status of the SR thereby contributing to the control of contractile force (Eisner et al. 2017; Gambardella et al. 2018). Accordingly, acute changes in the magnitude of I_CaL_ in- or decrease cardiac contractility and hence, cardiac output. The intracellular Ca^2+^ concentration not only controls contractility, but is also a potent regulator of gene expression (Dewenter et al. 2017). Long term changes in I_CaL_ magnitude are therefore considered an early step in the development of cardiac pathologies such as hypertrophy or heart failure and contribute to electrical and structural remodeling (Muth et al. 2001; Bosch et al. 2003; Chen-Izu et al. 2007; Zhang et al. 2016).

In addition to cardiac myocytes, L-type Ca^2+^ channels are widely expressed in various excitably and non-excitable tissues such as striated and smooth muscle, neurons, endocrine glands or fibroblasts, where they serve different and specific functions such as control of excitability, hormonal release or gene expression (Moosmang et al. 2003; Marcantoni et al. 2007; Calin-Jageman and Lee 2008; Zamponi et al. 2015). Tissue specific function of L-type Ca^2+^ channels originates to some extent from specific expression of four different variants of the pore-forming α-subunit (Ca_V_1.1–1.4) (Feng et al. 2018). Moreover, the α-subunits associate with additional β- and δ-subunits that modify expression levels and functional characteristics (Tseng et al. 2006). Of the four variants, Cav1.1 is mainly found in skeletal muscle and is crucial for contraction (Wu et al. 2017) whereas Cav1.2 and 1.3 show somewhat overlapping expression patterns and are located in adrenal chromaffin cells (Vandael et al. 2013), cardiac tissue (Bodi et al. 2005; Feng et al. 2018) and neuronal cells (Berger and Bartsch 2014). Furthermore, CaV1.3 is also found in the pancreas and the kidney and is essential for endocrine secretion (Barnett et al. 1995; Nitert et al. 2008) as well as in the cochlea contributing to auditory transduction (Brandt et al. 2003; Michna et al. 2003). CaV1.4 is mainly expressed in retinal cells and necessary for normal visual function (Lee et al. 2015). The cardiac variant of the α-subunit, Ca_V_1.2, is also expressed in smooth muscle and in neurons. Functional specificity of L-type Ca^2+^ channels not only originates from the different pore-forming α-subunits, but also from alternative splicing of the same α-subunit. In fact, in humans, the gene encoding Ca_V_1.2, *cacna1c*, is composed of at least 55 exons of which 19 undergo alternative splicing (Abernethy and Soldatov 2002; Tang et al. 2004). The corresponding gene in rats contains 52 exons with alternative splicing occurring in at least 11 of them (Tang et al. 2004; Wang et al. 2006). A number of studies have conferred functional differences of I_CaL_ in different tissues to alternative splicing. For example, activation and inactivation potentials of I_CaL_ in smooth muscle cells are shifted to more positive potentials presumably by expression of different splice variants of exons 1, 8 and 33 (Biel et al. 1991; Liao et al. 2005; Lipscombe & Andrade 2015). However, there is a large overlap in splice variant expression in different tissues. Exon 1a is exclusively expressed in cardiac myocytes whereas exon 1b is also found in smooth muscle cells and neurons (Snutch et al. 1991; Hofmann et al. 2014). A third variant of exon 1, exon 1c is also expressed in both cardiomyocytes and smooth muscle cells of resistance size cerebral arteries and is of particular importance for the smooth muscle phenotype of I_CaL_ (Cheng et al. 2007). It is therefore not surprising, that exon 1a-c expression quantitatively differs substantially between cardiac and smooth muscle cells. In cardiac myocytes, exon 1a accounts for ~ 80% of total exon 1 mRNA, exon 1b for ~ 5% and exon 1c for 15%. In contrast, in smooth muscle cells exon 1a is not expressed, exon 1b accounts for ~ 5% and exon 1c for 95% (Cheng et al. 2007).

Primary culture of isolated adult ventricular myocytes is a common and widely used tool to investigate non-acute effects (in the range of days) of drugs or hormones, signaling cascades or even altered gene expression on cardiomyocyte morphology or function. However, deprived of their natural environment primary cultured ventricular myocytes undergo substantial morphological and functional changes. They lose their rectangular shape, decrease in cell surface area, undergo alterations in the t-tubular system and the amplitude of their Ca^2+^ transient decreases as well as their contractility (Banyasz et al. 2008; Parameswaran et al. 2013; Seidel et al. 2019; Kim et al. 2020). With respect to I_CaL_, its amplitude has been found to decrease over days with slower inactivation and increased half-decay time of the Ca^2+^ transient (Banyasz et al. 2008; Kim et al. 2020). Regular pacing has been suggested to mitigate some negative effects of primary culture as it maintained contractility of isolated cardiac myocytes in primary culture (Berger et al. 1994). Moreover, regular pacing contributes to the stability of isolated ventricular tissue in culture (Fischer et al. 2019) and improves the differentiation of stem cells into ventricular cardiomyocytes (Nunes et al. 2013; Hirt et al. 2014; Ruan et al. 2016).

In the present study we investigated the effect of 24 h primary culture with and without pacing at 1 or 3 Hz on amplitude and kinetics of I_CaL_. We assessed total *cacna1c* mRNA expression and analyzed the expression of the three different variants of exon 1 to determine whether primary culture with or without pacing leads to alterations in isoform expression of the L-type Ca^2+^ channel α-subunit. We show that 24 h primary culture leads to a small but significant decrease in I_CaL_, but a substantially decrease in *cacna1c* total mRNA expression and in the expression of the cardiac isoform of exon 1 while exon 1b and exon 1c expression increased. Pacing with 1 or 3 Hz substantially decreased I_CaL_ amplitude and *cacna1c* total mRNA expression as well as the cardiac isoform of exon 1, whereas exon 1b and exon 1c expression remained unaltered.

## Methods

### Isolation of Myocytes

Cardiomyocytes were isolated from the left ventricular free wall of female Wistar rats (~ 220 g) as described previously (Wagner et al. 2010; Wacker et al. 2020). After induction of deep anesthesia by intraperitoneal injection of thiopental-sodium (100 mg kg ^− 1^ body mass), the heart was quickly excised and placed into cold (4 °C) Tyrode’s solution. Subsequently, the aorta was retrogradely perfused for 5 min with modified Tyrode’s solution containing 4.5 mM Ca^2+^ and 5 mM EGTA (~ 1 μM free Ca^2+^ concentration). The perfusion was continued for 19 min, recirculating 25 ml of the same solution containing collagenase (CLS type II, 160 U/ml, Biochrom KG, Berlin, Germany) and protease (type XIV, 0.6 U/ml, Sigma). Then, the heart was perfused for another 5 min with storage solution containing 100 μM Ca^2+^. The left ventricular free wall was separated from the rest of the heart und dissected into small tissue pieces. Tissue pieces were further minced and gently agitated to obtain single cardiomyocytes. Myocytes were stepwise adapted to physiological Ca^2+^ levels, transferred to six-well cell culture dishes containing M199 medium (Sigma) supplemented with 1 mg/mL bovine serum albumin (BSA, Roth) and penicillin/streptomycin (Biochrom) and stored at 37 °C in a water-saturated atmosphere containing 5% CO_2_. After a period of three hours, carbon-based pacing electrodes (C-Dish, IonOptix) were introduced into the six-well cultures dishes and 24 h pacing was initiated at 1 or 3 Hz using a bi-phasic stimulus of 7 ms duration. The pacing voltage stimulus was set to a level that ~ 90% of the myocytes contracted and ranged from 10.5 V to 14.5 V.

All experiments were performed in accordance with relevant guidelines and regulations and all experimental protocols were approved by the Regierung von Mittelfranken, license No: 621-2531.32-11/05.

### Patch-Clamp Technique

The ruptured-patch whole-cell configuration was used as described previously (Wagner et al. 2008). Myocardial cells were transferred into an elongated chamber (4 × 20 mm), mounted on the stage of an inverted microscope (Axiovert 40, Zeiss, Jena, Germany) and initially superfused with control solution. All experiments were performed at room temperature (22–25 °C). Patch pipettes were pulled from borosilicate glass (GC150-15, Clark Electromedical Instruments, Reading, UK) using a P-97 Puller (Sutter Instruments, Novato, CA, USA). Pipette resistance (RPip) was 1.5-5 MΩ. Currents were recorded using an EPC-10 amplifier (HEKA Elektronik, Lambrecht, Germany), controlled by PULSE-Software (HEKA Elektronik). Membrane capacitance (Cm) and series resistance (Rs) were calculated using the automated capacitance compensation procedure of the EPC-10 amplifier. Series resistance was in the range of ~ 5 MΩ, was not allowed to exceed 10 MΩ and was compensated by 85%. The reference electrode of the amplifier headstage was bathed in pipette solution in a separate chamber and was connected to the bath solution via an agar–agar bridge filled with pipette solution. Pipette potentials (V_Pip_) were corrected for liquid junction potentials at the bridge-bath junction. Whole-cell currents were low-pass filtered at 1 kHz and sampled at 5 kHz.

To assess I_CaL_, myocytes were clamped for 600 ms from the holding potential of − 90 mV to test potentials between − 60 mV and + 70 mV in steps of 10 mV. Na^+^ currents were inactivated by a prepulse of 70 ms to − 50 mV. In some experiments, if a residual Na^+^ current was still present, the prepulse voltage was decreased to V_Pip_ = − 45 mV. Basic cycle length was 3000 ms. I_CaL_ was quantified by subtracting the current at the end of the test pulse from the peak current. To determine activation kinetics, the slope conductance (G_CaL_) of I_CaL_ was calculated for each pipette potential (G_CaL_ = I_CaL_/(V_Pip_ – V_rev_) where V_rev_ is the apparent reversal potential of I_CaL_. G_CaL_ was then normalized to the maximal observed slope conductance recorded in each individual experiment and plotted vs. the pipette potential. Steady-state inactivation was determined by a two-step pulse protocol: a conditioning pulse of 600 ms duration from V_Pip_ =  − 90 mV to + 10 mV in steps of 10 mV was followed by a test pulse to V_Pip_ = 0 mV for 600 ms. The magnitude of I_CaL_ of each test pulse was normalized to I_CaL_ recorded at a conditioning potential of V_Pip_ = − 80 mV in each individual experiment and plotted vs. the pulse potential. Activation and steady-state inactivation were fitted using the Boltzmann equation. Recovery from inactivation was determined by two consecutive pulses to V_Pip_ = 0 mV of 600 ms duration, separated by a repolarization to V_Pip_ =  − 90 mV. The duration of the interval ranged from 5 to 1500 ms and increased by a factor 1.5 from step to step. The holding potential was V_Pip_ = − 90 mV, cycle length was 5000 ms. The magnitude of I_CaL_ recorded during the second voltage pulse was normalized to the magnitude of the first, plotted vs. the duration of the interval between the voltage pulses and the resulting curve was fitted using a mono-exponential equation.

### Quantitative Real Time PCR Analysis

Fresh or paced rat cardiomyocytes were centrifuged for 5 min at 1000 g and the pellets were immediately frozen at − 80 °C. RNA-isolation was performed using the NucleoSpin RNA-Kit from Macherey–Nagel, applying the manufactures protocol for cells and tissue. RNA-concentration of each sample was measured with the Denovix DS 11 + spectrophotometer. Using the QuantiTect reverse transcription kit from Quiagen, single-stranded cDNA was synthesized from 50 µg total RNA. Quantitative real time PCR was performed in the StepOnePlus cycler from Applied Biosystems, using the following sequence: 2 min at 50 °C and 2 min at 95 °C, then 40 cycles of 15 s at 95 °C and 1 min 60 °C and for the melt curve stage 15 s 95 °C, 1 min 60 °C and in 0.3 °C steps to 95 °C for 15 s. Analysis was performed using the Step One software, Microsoft Excel (Microsoft Corporation, Redmond, USA) and Prism (GraphPad, San Diego, USA). Specificity of primers was verified by PCR and gel electrophoresis followed by DNA sequencing. Samples were loaded in duplicates. The amplification efficiency for each gene was determined by a four-step serial dilution (five-fold) of the template. After correction for amplification efficiency, PCR results were normalized the arithmetic mean expression level of the three housekeeping genes (Gapdh, Hprt1 and Eef2. Fold change was calculated as a ratio of treated over control.

### Solutions and Primers

For the isolation of myocytes, modified Tyrode’s solution contained (in mM): NaCl 138, KCl 4, Glucose 10, NaH_2_PO_4_ 0.33, MgCl_2_ 1, HEPES 10, CaCl_2_ 0.9, EGTA 1, Insulin 10^–3^, titrated to pH 7.30 using NaOH. The same solution with 2 mM Ca^2+^ was used as bath solution for the patch-clamp and fluorescence imaging experiments. For cell digestion, collagenase (162.8 U/ml, CLSII, Biochrom AG, Berlin, Deutschland) and protease (0.54 U/ml, type XIV, Sigma-Aldrich GmbH, Steinheim, Deutschland) were added to modified Tyrode’s solution (10^–6^ M Ca^2+^). Storage solution contained (in mM): NaCl 130, NaH_2_PO_4_ 0.4, NaHCO_3_ 5.8, MgCl_2_ 0.5, CaCl_2_ 1, KCl 5.4, glucose 22, and HEPES 25, titrated to pH 7.40 with NaOH in the presence of 5% CO_2_ and supplemented with 1 mg ml^−1^ BSA. For recording Ca^2+^ currents, the pipette solution contained (in mM): CsCl 130, MgCl_2_ 5, EGTA 10, HEPES 10, Na_2_ATP 2, pH 7.20 with CsOH. The concentration of free ionized Ca^2+^ in this solution was calculated using the approach published by Schoenmakers et al. (Schoenmakers et al. 1992) and was ~ 0.4 nM (pCa ~ 9.4).

**Table:** 

Gene	Primer 5′- 3′
Exon 5	fw: gagagctttccgtgtgcttc rev: ccttgatgatggagttcagga
Exon 1a	fw: aaggtggtacacgaagctcaa rev: cgtgggctcccatagttg
Exon 1b	fw: caatggtcaatgaaaacacga rev:ggctcccatagttggaacct
Exon 1c	fw: ctaggaagtgcagtaatgaccgtg rev: gttggaacatgcacagtccagag
Gapdh	fw: tgggaagctggtcatcaac rev: gcatcaccccatttgatgtt
Hprt1	fw: cccagcgtcgtgattagtga rev: tcgagcaagtctttcagtcct
Eef2	fw: aggccgccatgggtattaag rev: aaggcatagaagcggccttt

### Data Analysis and Statistics

Patch Clamp data were analyzed using the PULSE-FIT software (HEKA Elektronik, Lambrecht/Pfalz, Germany), IGOR Pro (WaveMetrics, Lake Oswego, USA), and Microsoft Excel as described previously (Foltz et al. 2012). Data are given as mean ± SEM. Statistical significance was evaluated by unpaired Student's t test when two groups were compared or one-way ANOVA followed by Newman-Keuls test when more than two groups were compared using Prism. p < 0.05 was considered statistically significant.

## Results

### Primary Culture and Pacing with 1 and 3 Hz Decreases L-type Ca^2+^ Current Amplitude

**Fig. 1:**
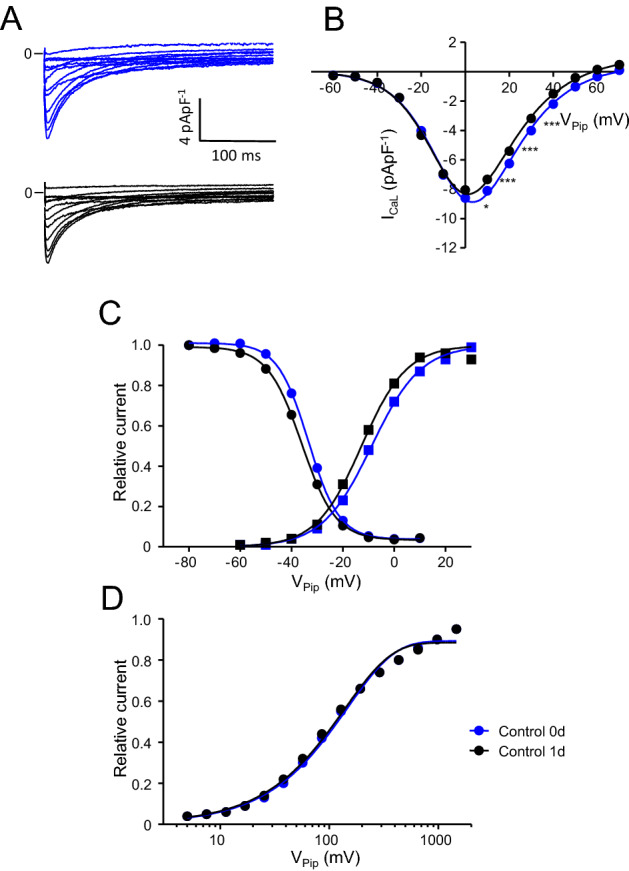
Primary culture (24–32 h) decreases I_CaL_ density. **A** representative whole-cell current traces of I_CaL_ recorded from freshly isolated myocytes (blue traces, ‘Control 0d’) and myocytes incubated for 24–32 h without pacing (black traces, ‘Control 1d’). **B** average current–voltage relations of currents similar to those shown in (**A**). I_CaL_ was quantified by subtracting the peak current from the current at the end of the voltage pulse (at 600 ms). **C** activation (circles) and steady-state inactivation (boxes) of I_CaL_ recorded from both groups. **D** recovery from inactivation of I_CaL_ recorded from both groups. For details regarding pulse protocols see methods section. Note that error bars are smaller than the symbols. 56 ≤ n ≤ 111. *p < 0.05, ***p < 0.001, Control 0d vs. Control 1d

It has been reported that L-type Ca^2+^ current (I_CaL_) of freshly isolated rat left ventricular myocytes decreases (Banyasz et al. 2008), remains stable (Yang et al. 2012) or increases (Ellingsen et al. 1993) during 24 h of primary culture. We therefore first investigated I_CaL_ amplitude directly after cell isolation (0–8 h) and after 24 h (24–32 h) of primary culture to investigate if and to what extent 24 h primary culture affects I_CaL_ amplitude and kinetics under our experimental conditions. Figure 1A displays representative current traces obtained from a myocyte directly after isolation (blue) and after 24 h incubation (black). I_CaL_ traces appear quite similar. Figure 1B summarizes similar experiments and shows average current–voltage (I–V) relations, recorded from myocytes directly after isolation (blue) and after 24 h incubation (black). On average I_CaL_ decreased to a small (~ 10%) but significant extent at command potentials from V_Pip_ = 10 to 40 mV. Peak I_CaL_ recorded at V_Pip_ = 0 mV decreased from − 9.2 ± 0.3 pApF^−1^ (n = 67) to − 8.3 ± 0.3 pApF^−1^ (n = 62, p = 0.057). To address inactivation of I_CaL_ a biexponential function was fitted to the current decay at V_Pip_ = 0 mV, hence yielding two time constants of inactivation, τ_1_ and τ_2_ (Wagner et al. 2008). Table 1 summarizes I_CaL_ inactivation. Neither τ_1_ or τ_2_, nor their relative contribution to total inactivation was affected by 24 h incubation of the myocytes. Figure 1C shows average steady-state activation and steady-state inactivation curves obtained from myocytes directly after isolation (blue) and after 24 h incubation (black). 24 h incubation significantly shifted steady-state activation and inactivation to more negative potentials from − 8.6 ± 0.5 mV (n = 68) to − 12.8 ± 0.6 mV (n = 62, p < 0.001) and from − 33.4 ± 0.4 mV (n = 111) to − 36.4 ± 0.5 mV (n = 72, p < 0.001), respectively and led to a small increase in the slope factor of steady-state inactivation from − 5.4 ± 0.1 mV (n = 111) to − 6.2 ± 0.1 mV (n = 72, p < 0.001) (detailed parameters are summarized in Table 2). Recovery from inactivation (Fig. 1D) was not significantly affected by 24 h incubation and followed a monoexponential characteristic with an average time constant of 154.1 ± 4.8 ms (n = 77) in freshly isolated myocytes and 148.9 ± 7.1 mV (n = 56, n.s.) in myocytes after 24 h incubation.

**Table 1:** Inactivation time constant of I_CaL_ after 24–32 h primary culture

	n	τ 1 (ms)	τ 2 (ms)	% τ 1 (%)	% τ 2 (%)
Control 0d	66	20.1 ± 0.7	105 ± 2.9	67.4 ± 0.7	32.6 ± 0.7
Control 1d	60	20.2 ± 0.7	108 ± 3.0	67.0 ± 0.7	33.0 ± 0.7

*τ 1 and τ 2* inactivation time constants of I_CaL_ obtained by bi-exponentially fitting of current decay at V_Pip_ = 0 mV, *% τ 1 and % τ 2* contribution of τ 1 and τ 2 to total inactivation (values are given in % of total inactivation), *n* number of myocytes

**Table 2:** Steady-state activation and inactivation parameters of I_CaL_ after 24–32 h primary culture

	V_50 act_ (mV)	*k*_act_ (mV)	n_act_	V_50 inac_ (mV)	*k*_inac_ (mV)	n_inac_
Control 0d	− 8.6 ± 0.4	9.3 ± 0.2	68	− 33.4 ± 0.4	− 5.4 ± 0.1	111
Control 1d	− 12.8 ± 0.6*	8.1 ± 0.2	62	− 36.4 ± 0.5***	− 6.2 ± 0.1***	72

*V*_*50 act*_ voltage at which half-maximal steady-state activation was observed, *k*_*act*_ slope factor of the steady-state activation curve, *V*_*50 inac*_ voltage at which half-maximal steady-state inactivation was observed, *k*_*inac*_ slope factor of the steady-state inactivation curve, *n*_*act*_ number of myocytes for steady-state activation, *n*_*inac*_ number of myocytes for steady-state inactivation

*p < 0.05, ***p < 0.001 vs. Control 0d

**Fig. 2:**
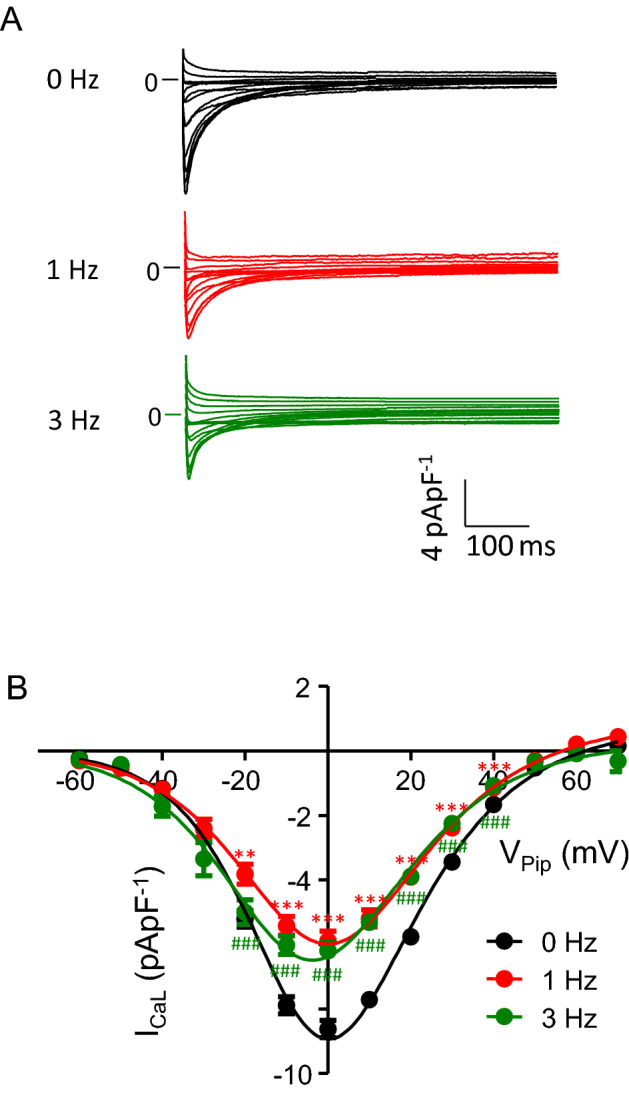
Pacing at 1 or 3 Hz decreases I_CaL_ density. **A** representative whole-cell current traces of I_CaL_ recorded from myocytes incubated for 24–32 h without pacing (black traces, ‘0 Hz’) and with pacing at 1 Hz (red traces, ‘1 Hz’) and at 3 Hz (green traces, ‘3 Hz’). **B** average current–voltage relations of currents similar to those shown in (**A**). I_CaL_ was quantified by subtracting the peak current from the current at the end of the voltage pulse (at 600 ms). Note that most error bars are smaller than the symbols. 48 ≤ n ≤ 76. **p < 0.05, ***p < 0.001, 1 Hz vs. 0 Hz. ^###^p < 0.001, 3 Hz vs. 0 Hz

**Fig. 3:**
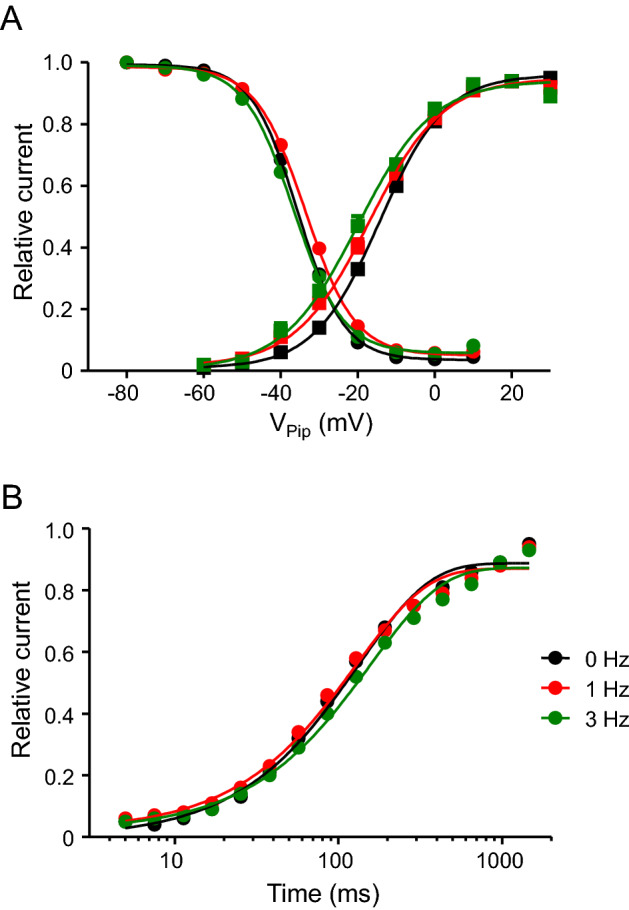
Pacing at 1 or 3 Hz alters I_CaL_ kinetics. **A** activation (circles) and steady-state inactivation (boxes) of I_CaL_ recorded from myocytes incubated for 24–32 h without pacing (black, ‘0 Hz’) and with pacing at 1 Hz (red, ‘1 Hz’) and at 3 Hz (green, ‘3 Hz’). **B** recovery from inactivation of I_CaL_ recorded from the groups mentioned above. For details regarding pulse protocols see methods section. Note that most error bars are smaller than the symbols. 50 ≤ n ≤ 71

Next, we investigated, whether continuous pacing over 24 h at a low rate of 1 Hz or at a higher rate of 3 Hz prevented the observed decrease in I_CaL_. Figure 2 shows representative current traces obtained from left ventricular myocytes incubated for 24 h under control conditions (Fig. 2A, 0 Hz), at a pacing rate of 1 Hz and 3 Hz. 24 h pacing at 1 or 3 Hz led to a substantial decrease in I_CaL_ amplitude compared to non-paced control myocytes. Figure 2B shows average current–voltage (I–V) relations obtained from experiments similar to those shown in Fig. 2A. On average, pacing at 1 Hz decreased I_CaL_ amplitude by 31% from − 8.6 ± 0.3 pApF^−1^ (n = 74) to 5.9 ± 0.3 pApF^−1^ (n = 56, p < 0.001, V_Pip_ = 0 mV). A similar reduction in I_CaL_ amplitude (6.2 ± 0.2 pApF^−1^, n = 48, p < 0.001, V_Pip_ = 0 mV) was observed after pacing with 3 Hz. To address the question whether pacing altered single channel behavior of L-type Ca^2+^ channels, kinetic analysis was performed. Inactivation time constants of I_CaL_ are summarized in Table 3. 24 h pacing at 1 Hz led to small, but significant increase in τ_1_ and τ_2_ from 18.3 ± 0.5 ms to 20.7 ± 0.8 ms (p < 0.05; V_Pip_ = 0 mV) and from 103 ± 2.3 ms to 118 ± 3.2 ms (p < 0.001), respectively. Moreover, the contribution of the short time constant τ_1_ to total inactivation decreased from 68.5 ± 0.6% to 65.3 ± 0.6% (p < 0.001). Similar results were obtained at a pacing rate of 3 Hz. Steady-state activation and steady-state inactivation are summarized in Fig. 3A. In control myocytes (0 Hz, black) half-maximal activation was observed at V_50_ = -15.3 ± 0.4 mV (n = 71), while pacing with 1 Hz (red) and 3 Hz (green) significantly shifted half-maximal activation to more negative potentials of V_50_ = − 17.5 ± 0.7 mV (n = 54, p < 0.05) and V_50_ = − 19.3 ± 1.0 mV (n = 50, p < 0.001), respectively. The slope factor was not affected and averaged ~ 7.2 mV (for detailed values see Table 4). In contrast, steady-state inactivation was only marginally affected by 1 Hz pacing with V_50_ = − 35.5 ± 0.5 mV (n = 74, 0 Hz) and V_50_ = − 33.6 ± 0.5 mV (n = 66, 1 Hz, p < 0.05). Figure 3B summarizes the recovery from inactivation. Pacing at 1 Hz slightly but significantly shortened I_CaL_ recovery from τ_rec_ = 140 ± 3.6 ms (n = 71) to τ_rec_ = 128 ± 3.5 ms (n = 67, p < 0.05) while 3 Hz pacing prolonged it to τ_rec_ = 154 ± 6.3 ms (n = 51, p < 0.05).

**Table 3:** Inactivation time constant of I_CaL_ after pacing at 1 or 3 Hz

	n	τ 1 (ms)	τ 2 (ms)	% τ 1 (%)	% τ 2 (%)
0 Hz	73	18.3 ± 0.5	103 ± 2.3	68.5 ± 0.6	31.5 ± 0.6
1 Hz	57	20.7 ± 0.8*	118 ± 3.2***	65.3 ± 0.6***	34.7 ± 0.6***
3 Hz	52	20.7 ± 0.8*	118 ± 2.7***	63.6 ± 0.6***	36.4 ± 0.6***

*τ 1 and τ 2* inactivation time constants of I_CaL_ obtained by bi-exponentially fitting of current decay at V_Pip_ = 0 mV; *% τ 1 and % τ 2* contribution of τ 1 and τ 2 to total inactivation (values are given in % of total inactivation), *n* number of myocytes

*p < 0.05, ***p < 0.001 vs. 0 Hz

**Table 4:** Steady-state activation and inactivation parameters of I_CaL_ after pacing at 1 or 3 Hz

	V_50 act_ (mV)	*k*_act_ (mV)	n_act_	V_50 inac_ (mV)	*k*_inac_ (mV)	n_inac_
0 Hz	− 15.3 ± 0.4	7.7 ± 0.2	71	− 35.5 ± 0.5	− 5.3 ± 0.1	74
1 Hz	− 17.5 ± 0.7*	8.2 ± 0.4	54	− 33.6 ± 0.5	− 6.0 ± 0.1	66
3 Hz	− 19.3 ± 1.0***	7.7 ± 0.3	50	− 36.6 ± 0.5	− 5.8 ± 0.1	57

*V*_*50 act*_ voltage at which half-maximal steady-state activation was observed, *k*_*act*_ slope factor of the steady-state activation curve, *V*_*50 inac*_ voltage at which half-maximal steady-state inactivation was observed, *k*_*inac*_ slope factor of the steady-state inactivation curve, *n*_*act*_ number of myocytes for steady-state activation, *n*_*inac*_ number of myocytes for steady-state inactivation

*p < 0.05, ***p < 0.001 vs. 0 Hz

### Incubation and Stimulation Alter mRNA Expression of Exon 1 and Exon 5 of Cacna1c

**Fig. 4:**
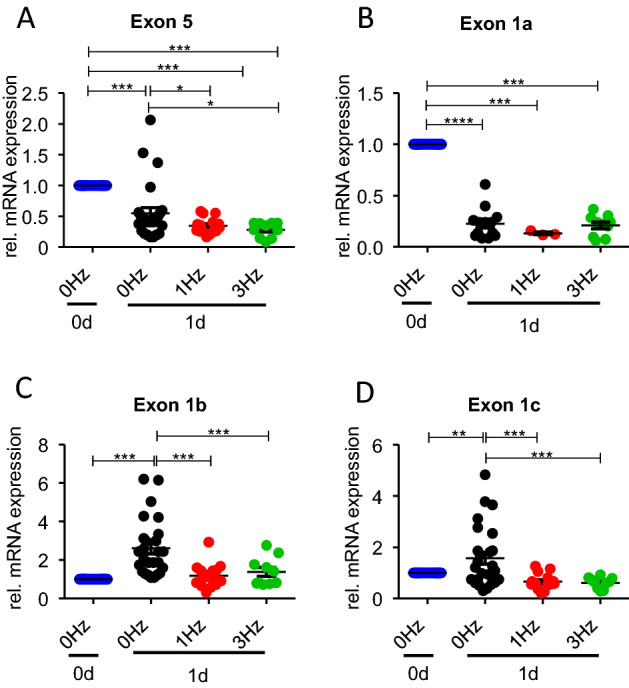
Effects of pacing on mRNA expression of total *cacna1c* (exon 5) and exon 1 splice variants. **A** mRNA expression of exon 5 of freshly isolated myocytes (blue, 0 Hz, 0d) and of myocytes incubated for 24 h without pacing (black, 0 Hz, 1d) and with pacing at 1 Hz (red, 1 Hz, 1d) and at 3 Hz (green, 3 Hz, 1d). 10 ≤ n ≤ 16. **B** mRNA expression of exon 1a in the same groups as in (**A**). 3 ≤ n ≤ 16. **C** mRNA expression of exon 1b in the same groups as in (**A**). 10 ≤ n ≤ 16. **D** mRNA expression of exon 1c in the same groups as in (**A**). 10 ≤ n ≤ 16. mRNA expression was normalized to the level of freshly isolated myocytes (blue, 0 Hz, 0d) in each individual experiment. *p < 0.05, **p < 0.05, ***p < 0.001

In an independent set of experiments, we then investigated the effect of 24 h incubation alone and of 24 h pacing at 1 Hz and 3 Hz on mRNA expression of the pore-forming α-subunit of the cardiac L-type Ca^2+^ channel, *cacna1c*. After cell isolation, a fraction of myocytes was directly frozen for further processing, whereas the rest was either cultured under control conditions for 24 h (0 Hz) or subjected to continuous pacing for 24 h at 1 Hz or 3 Hz, after which the myocytes were frozen. For analysis of total *cacna1c* mRNA expression, we used specific intron-overspanning primers for exon 5, which is present in every splice variant and is therefore a useful marker for total *cacna1c* mRNA. Figure 4A summarizes exon 5 mRNA expression assessed by quantitative real-time RT-PCR and normalized to the expression level of exon 5 mRNA in freshly isolated myocytes. Incubation for 24 h under control conditions (0 Hz) led to a significant decrease in exon 5 mRNA expression to 54.9 ± 8.9% (n = 26; p < 0.001) of the level of freshly isolated cardiomyocytes. Pacing at 1 Hz and 3 Hz led to a further decrease in exon 5 mRNA expression down 34.5 ± 3.1% (n = 16; p < 0.001) and 28.2 ± 3.6% (n = 10; p < 0.001), respectively. These results are qualitatively consistent with the decrease in I_CaL_ amplitude observed after 24 h incubation alone and the further decrease in I_CaL_ induced by pacing with 1 Hz or 3 Hz. We then asked if primary culture and pacing not only affect the total amount of *cacna1c* mRNA but also alter its isogene composition. Since especially splice variants of exon 1 are differentially expressed in cardiac myocytes, we addressed the expression of its three splice variants exon 1a, 1b and 1c using specific primers (see methods). Figure 4B shows that the expression of the cardiac specific variant of exon 1 decreased by 24 h primary culture under control conditions, a result which is similar to the decrease in expression of exon 5. Pacing at 1 or 3 Hz did not further alter the decrease in expression of exon 1a. In contrast, the expression of exon 1b and 1c (Fig. 4C, D), which is normally considerably lower than exon 1a expression in cardiac myocytes, significantly increased during 24 h incubation. This increase, however, was prevented by pacing at 1 or 3 Hz.

## Discussion

In the present study we observed a small but significant decrease in I_CaL_ density of ~ 10% in isolated left ventricular myocytes after 24–36 h of primary culture. In previous reports, a substantial decrease (Mitcheson et al. 1996; Banyasz et al. 2008), an increase (Ellingsen et al. 1993) or no change (Yang et al. 2012) in I_CaL_ density in isolated cardiac myocytes after 24 h in primary culture have been reported. It is noteworthy that I_CaL_ density in primary culture is affected by hormones or growth factors often added to the culture medium such as corticosteroids (Bénitah and Vassort 1999; Wagner et al. 2008), thyroid hormones (Sunagawa et al. 2005; Yu et al. 2012), or insulin (Wacker et al. 2020). Therefore, culture conditions are important when interpreting spontaneous alterations of I_CaL_ density in primary culture. For example, Ellingsen et al. observed an increase in I_CaL_ density after 24 h of primary culture (Ellingsen et al. 1993) that was probably caused by the inclusion of T3 and insulin in the culture medium. Yang et al. (Yang et al. 2012) observed a rather constant I_CaL_ density over a period of up to three days in isolated rabbit ventricular cardiomyocytes. However, they included 5% fetal bovine serum in the culture medium which contains, albeit at low concentrations, corticosteroids, IGF-1 and insulin, all well known to increase I_CaL_ density in primary culture (Bénitah and Vassort 1999; Wagner et al. 2008; Wacker et al. 2020). Using serum-free conditions without the addition of growth factors or hormones, Banyas et al. (Banyasz et al. 2008) observed a substantial decrease of I_CaL_ of ~ 30% in isolated rat left ventricular cardiomyocytes within 24 h. In contrast to our study, they added taurine, creatine and l-carnitine to the culture medium which might have altered I_CaL_. Taurine, for example, has been shown to in- or decrease I_CaL_ depending on the intracellular Ca^2+^ concentration in ventricular cardiomyocytes of the guinea pig (Satoh and Horie 1997).

The mechanisms underlying the decline of I_CaL_ density in primary culture are unclear at the present. Banyas et al. reported a decrease in density of the t-tubular system after 24 h of primary culture, which could account for a decrease in I_CaL_, since a substantial fraction of L-type Ca^2+^ channels is located in the t-tubular system (Banyasz et al. 2008). In a previous report, however, we did not observe a decrease in density of the t-tubular system after 24 h of primary culture under identical experimental conditions (Seidel et al. 2019), suggesting that other mechanisms underlie the small decrease in I_CaL_ density observed in the present study. Consistent with the decrease in I_CaL_ density we observed a decrease in total *cacna1c* mRNA expression after 24 h in unstimulated primary culture. Since the number of L-type Ca^2+^ channels in the plasma membrane is a function of the rate of insertion of newly synthesized channels into the membrane and the net rate of retrieval of L-type Ca^2+^ channels from the membrane (Westhoff and Dixon 2021), a decrease in total *cacna1c* mRNA expression could lead to decrease in the number of L-type Ca^2+^ channels in the plasma membrane within 24–36 h, provided that the channel half-life in the membrane is in an appropriate range. The half-live of L-type Ca^2+^ channels in the plasma membrane of cardiac myocytes is subject to complex regulation involving endosomal recycling of channels as well as pools of readily insertable CaV1.2 that form an internal reservoir that can be inserted in the plasma membrane in certain conditions (Ito et al. 2019; del Villar et al. 2021). Using nocodazole to stop trafficking of newly synthesized channels to the plasma membrane, channel half-life was estimated to be above 20 h in an immortalized atrial cell line (AT-1) (Conrad et al. 2018). In contrast, in freshly isolated mouse ventricular myocytes half-life was somewhat above 2 h only (del Villar et al. 2021). It is therefore safe to assume that the decrease in total *cacna1c* mRNA expression after 24–36 h incubation caused the small decrease in I_CaL_ density.

In the present study, 24–36 h incubation had no effect on I_CaL_ inactivation and recovery from inactivation, however, steady-state activation and steady-state inactivation were shifted by a minor degree of 3–4 mV to more negative potentials. In another study using rat cardiomyocytes, no effect of primary culture on I_CaL_ kinetics was observed (Banyasz et al. 2008), however, our study included more than 60 myocytes per group, hence, the power to detect even minor differences in I_CaL_ kinetics was substantially larger in our study than e.g. in the study by Banyas et al., which compared 7 myocytes per group. Similar results were obtained in rabbit cardiomyocytes (Mitcheson et al. 1996). The observed shift in steady-state activation and inactivation could result from several causes. Alterations in L-type channel phosphorylation at the N-terminal domain by Protein Kinase C for example (McHugh et al. 2000; Yang et al. 2005) during primary culture could account for the observed change. Alternatively, the composition of the underlying L-type Ca^2+^ channels might have changed. Indeed, we observed an increase in mRNA expression of the non-predominant exon 1b and exon 1c after 24 h incubation. Since exon 1b and exon 1c are also expressed in smooth muscle cells (Cheng et al. 2007) and potentially also in fibroblasts (Chen et al. 2010), the increase in exon 1b + c expression during 24 h primary culture might be secondary to proliferation of smooth muscle cells or fibroblast that may be present in the cardiomyocyte culture. However, in control experiments we found that the mRNA expression of vimentin and DDR2, both markers for smooth muscle cells and fibroblasts (Camelliti et al. 2005), decreased during 24 h primary culture (data not shown). This suggests that the increase in mRNA expression of exon 1b + c after 24 h incubation most likely results from a decrease in their expression in cardiomyocytes. While an early study did not detect significant differences in steady-state activation or inactivation between the cardiac and the vascular variant of Cav1.2 (Hu and Marban 1998), Bartels et al. (Bartels et al. 2018) more recently showed in an elegant study, that replacing exon 1a by exon 1b leads to a shift of steady-state activation and inactivation towards more negative potentials by 3–4 mV. Since L-type Ca^2+^ channels composed of exon 1c display similar steady-state activation and inactivation as Ca^2+^ channels containing exon 1b (Cheng et al. 2007), one could speculate that the small shift in steady-state activation and inactivation potentials originates from an increased contribution of Ca^2+^ channels containing exon 1b or exon 1c instead of exon 1a.

Taken together, we have identified serum-free culture conditions under which I_CaL_ density remains rather constant and is therefore suitable for experiments addressing medium to long term effects over a period of 24–36 h on I_CaL_.

Pacing at 1 or 3 Hz for 24–36 h substantially decreased I_CaL_ density as well as total *cacna1c* mRNA expression compared to non-paced controls incubated under identical conditions. This is in line with observations in a canine model of atrial fibrillation induced by rapid pacing for 1, 7, and 42 days in which a continuous decrease in I_CaL_ density in atrial cardiomyocytes was observed over the period investigated (Yue et al. 1997) as well as a decrease in mRNA expression of *cacna1c* (Yue et al. 1999). Similar results were obtained in rabbits upon rapid ventricular pacing for 24 h which led to a decrease in I_CaL_ density and *cacna1c* mRNA expression (Bosch et al. 2003). Moreover, 3 Hz pacing of isolated canine atrial myocytes for 24 h decreased I_CaL_ density by 55%, while 1 Hz pacing did not (Satin et al. 2011). In contrast, in isolated rat cardiac myocytes, an increase in I_CaL_ has been reported after 48 h of 3 Hz pacing (Berger et al. 1994). However, in this study, myocytes were incubated with different hormones and substances known to alter I_CaL_ such as T3, insulin, creatine and taurine and, using identical culture conditions, the same group also observed an increase in I_CaL_ by incubation alone (Ellingsen et al. 1993). It is therefore conceivable that the increase in I_CaL_ they observed is at least related to the presence of the aforementioned substances.

Pacing of isolated cardiomyocytes in primary culture resembles the regular electrical excitation the myocytes experience in vivo. The contractile response, however, differs. Individual myocytes in a culture dish contract freely without any extra load (so-called unloaded cell shortening), so they only generate the force necessary to alter their shape from the relaxed to the contracted form. This force is far less compared to the force developed in vivo. Therefore, one could consider the decrease in I_CaL_ density as a response to unloaded regular contractions. A comparable in vivo condition has been achieved in rats by transplanting a donor heart to the abdominal aorta of a recipient animal, where it receives coronary perfusion and contracts regularly without ventricular filling or a stroke volume over a long time (up to several weeks). Unloaded hearts develop a significant atrophy and undergo several changes in electrophysiology, Ca^2+^ cycling, contractility and gene expression (Schwoerer et al. 2008, 2013; El-Armouche et al. 2010). However, I_CaL_ density was not affected (Ibrahim et al. 2010) after 2 weeks or even increased (Schwoerer et al. 2008) after four weeks of cardiac unloading, suggesting that the decrease in I_CaL_ density caused by pacing of isolated myocytes in primary culture may not directly be related to cardiac atrophy. Instead, there is evidence, that pacing of isolated myocytes at 3 Hz might even lead to a hypertrophic response as it increased the rate of phenylalanine incorporation, total cellular protein content, and cell size (Kaye et al. 1996). Depending on etiology, duration and other factors, cardiac hypertrophy can exert different effects on I_CaL_ including an increase, no change or a decrease of I_CaL_ density (Volk and Ehmke 2002). Nevertheless, a more recent study showed that a decrease in I_CaL_ density can lead to hypertrophy (Goonasekera et al. 2012). Taken together, the observed decrease in I_CaL_ density in the present study can be considered as part of a hypertrophic response induced by pacing, however, further experiments need to address this hypothesis.

Cardiomyocyte pacing could also indirectly alter I_CaL_ density via affecting the density of the t-tubular system. Loss or alterations in the t-tubular system are a common phenomenon in heart failure with reduced ejection fraction, independent of its etiology, and the t-tubular system has the potential to recover, if the noxious stimulus is removed (Setterberg et al. 2021). Interestingly, pacing rate and rhythm might also affect the t-tubular system. T-system recovery has been observed in a canine model of dysynchronous heart failure, when regular biventricular pacing was applied (Sachse et al. 2012). Moreover, the regularity of pacing might also be important, since heart failure associated alterations in the t-tubular system could be reversed by reinstating heart rate variability in a goat model of heart failure (Shanks et al. 2022). We therefore cannot exclude that potential pacing-induced changes of the t-tubular system might have contributed to the observed changes in I_CaL_.

Pacing with 1 or 3 Hz led to minor alterations in I_CaL_ kinetics. Both, the fast and the slow inactivation time contents τ_1_ and τ_2_ increased. Since especially the fast inactivation of I_CaL_ is mediated by Ca^2+^ entering through the open channels (Hadley and Hume 1987), the increase of τ_1_ might be secondary to the substantial decrease in I_CaL_ density after 24–32 h of 1 or 3 Hz pacing. Moreover, pacing significantly shifted steady-state activation to more negative potentials while steady-state inactivation was only marginally affected. Similar to the effect of incubation alone, this could be a result of a relative decrease of the expression of exon 1a compared to exon 1b and exon 1c observed after pacing. Recovery from inactivation did not follow a clear pattern, pacing at 1 Hz led to a mild decrease while pacing at 3 Hz lead to a mild increase in the recovery time constant.

## Data Availability

The datasets generated during and/or analysed during the current study are available from the corresponding author on reasonable request.
